# The Brazilian Dilemma: Increased Scientific Production and High Publication Costs during a Global Health Crisis and Major Economic Downturn

**DOI:** 10.1128/mBio.00907-16

**Published:** 2016-06-28

**Authors:** Marcio L. Rodrigues, Carlos M. Morel

**Affiliations:** a Fundação Oswaldo Cruz (Fiocruz), Centro de Desenvolvimento Tecnológico em Saúde (CDTS), Rio de Janeiro, Brazil; b Instituto de Microbiologia Professor Paulo de Góes, Universidade Federal do Rio de Janeiro, Rio de Janeiro, Brazil

## GUEST EDITORIAL

Between 2002 and 2014, Brazil experienced tremendous growth in its production of scientific knowledge as a consequence of major increases in research funding (1). The economic performance of Brazil steadily improved during this period, which resulted in record growth in 2007 and 2008 (2). Large capital inflows into the scientific infrastructure contributed to a rapid modernization of labs that expanded capacity in many areas of science throughout Brazil (3). According to the SCImago database (http://www.scimagojr.com), in 1996 Brazil was ranked 21st among all countries worldwide in the publication of citable documents (8,652 articles, reviews, and conference papers) across all areas of science. The most recent SCImago analysis (2015) ranked Brazil in 13th place based on the generation of 61,122 citable documents, which represents a 7.0-fold surge in productivity.

Over the last 2 years, Brazil has been immersed in a major political and economic crisis that has directly affected funding for science. Furthermore, science in Brazil, as well as other developing countries, is especially susceptible to rising currency exchange rates that erode the purchasing power of available funds, since most reagents and equipment are imported from Europe and the United States. This also includes publication fees, which are most frequently paid in U.S. dollars. According to the Brazilian Central Bank, during the peak of economic growth in 2008, one U.S. dollar corresponded to approximately 1.7 Brazilian real. In April 2016, the exchange rate for the Brazilian real to U.S. dollar was approximately 3.5 to 1. This 100% increase in the exchange rate, combined with the decrease in science funding, has severely affected Brazilian scientists and their ability to conduct science, from the importation of consumables and equipment to the payment of publication fees.

Publication fees in the mainstream open-access journals vary considerably. At the current currency exchange rates, the majority of individual funding programs available to support the research program of a single Brazilian principal investigator ranges from $8,500 to $35,000 to cover a 3-year period (4). A conservative estimate indicates that the publication of one paper per year in open-access journals charging $1,500 during these 3 years would consume approximately 10 to 50% of a Brazilian grant, just for publication fees. Since Brazil is no longer included in the World Bank’s lists of low-income economies or lower-middle-income economies due to its economic growth, Brazilian authors are precluded from qualifying for publication cost waivers.

It is clear from a financial perspective that the costs of disseminating research findings must be paid by someone (5). Publication charges to authors are rooted in the business models of the 1960s and have been the topic of intense discussions ever since. In 1969, for instance, a 10 to 25% reduction in U.S. grants stimulated discussions about the decision between postponing publication and begging off from paying charges “by effectively declaring as a pauper” (6). Almost 5 decades later, Brazilian scientists face a similar dilemma, since successful grant applications, positive performance evaluations, and career progression depend on publishing—publish or perish.

From a global health perspective, Brazilian researchers, clinicians, and epidemiologists are often on the front line of emerging threats. Their scientific endeavors were instrumental in unveiling the recent epidemic of Zika virus infections and its relationship to microcephaly (7–11), which has led the World Health Organization to declare a Public Health Emergency of International Concern (PHEIC) (12). However, the cost of page charges in high-impact, open-access journals caused delays in publication and the dissemination of pivotal data as authors searched for funds that, when unavailable, resulted in resubmissions to international journals that do not charge to publish and often impose more-restricted access. As the Zika virus epidemic spreads across the developing world into the first world, the importance of local scientists and their contributions cannot be overemphasized. Considering this current scenario, discussions are urgently needed between Brazilian scientists, global decision makers, and scientific publishers. Concrete actions must be taken to overcome the existing financial barriers to information vital to combating current and future health threats. This is not solely a Brazilian issue.
